# The Microbial Diversity and Antimicrobial Susceptibility Profile Underlying Diabetic Foot Osteomyelitis: A Retrospective Study Conducted in North Queensland, Australia

**DOI:** 10.1177/24730114241281503

**Published:** 2024-09-30

**Authors:** Nandini Kulasegaran, Venkat Vangaveti, Robert Norton, Usman Malabu

**Affiliations:** 1 University of Queensland, Brisbane, Queensland, Australia; 2 College of Medicine and Dentistry, James Cook University, Townsville, Queensland, Australia; 3 Townsville Institute of Health Research and Innovation, Townsville University Hospital, Townsville, Queensland, Australia; 4 Translational Research in Endocrinology and Diabetes, College of Medicine and Dentistry, James Cook University, Douglas, Queensland, Australia; 5 Department of Microbiology, Townsville University Hospital, Townsville, Queensland, Australia; 6 Faculty of Medicine, University of Queensland, Brisbane, Queensland, Australia; 7 Department of Endocrinology & Diabetes, Townsville University Hospital, Townsville, Queensland, Australia

**Keywords:** diabetic foot osteomyelitis, diabetic foot infections, microbial profile, microbiological profile, microbial diversity, antibiotic susceptibility, antibiotic sensitivity

## Abstract

**Background:**

Diabetic foot osteomyelitis (DFO) commonly occurs secondary to ulcerations of the skin. Empirical antibiotic agents are a key element of treatment and their use is dependent on local knowledge of the microbial spectrum of diabetic foot infections. This study aimed to retrospectively analyze the local microbiological profile, including bacterial culture/sensitivity results of DFO, and compare findings with literature. This study also aimed to review the concordance of microbiology results with national guidelines for the future treatment of DFO.

**Methods:**

A retrospective review of clinical records was performed on patients who presented to the high-risk foot clinic, Townsville University Hospital, between 2018 and 2022. All patients older than 18 years and diagnosed with DFO were included. Our exclusion criteria included all other foot presentations, including trauma, vasculitis, and neoplasms.

**Results:**

On the basis of the inclusion and exclusion criteria, 124 patients with DFO were selected. Most patients in the cohort were males (70.2%), non-Indigenous (68.5%), aged 50-69 years (55.6%), and with elevated HbA_1c_ levels (>8.6). Chronic kidney disease (39.5%) and ischemic heart disease (41.9%) were common comorbidities. Of the pertinent microbial results, *Staphylococcus aureus* (~76%) was the most commonly isolated Gram-positive organism. Gram-positive bacteria were significantly increased in the elderly population with DFO (*P* < .05). All methicillin-resistant *S aureus* isolates were vancomycin- and cotrimoxazole-sensitive. *Pseudomonas aeruginosa* was the predominant Gram-negative organism isolated (39.3%). *P aeruginosa* exhibited low sensitivity to ciprofloxacin.

**Conclusion:**

This study has enhanced our understanding of the various microbial species underlying DFO at our center and may be generalizable.

**Level of Evidence:**

Level IV, retrospective case series.

## Introduction

Diabetic foot ulcers (DFUs) are formed by a complex interplay of poorly managed glycemic control, neuropathy, and peripheral vascular disease.^6^ Infected DFUs can lead to osteomyelitis in >20% of patients with moderate infections and can also lead to amputation.^6,17,25,27,28,34,38^ Of these, Aboriginal and Torres Strait Islander people experience a disproportionately increased rate of major amputations secondary to diabetic foot infections (DFIs) compared with non-Indigenous people.^11^

According to the Australian guidelines for diabetes-related foot disease, a comprehensive diagnostic approach, including clinical signs of infection, probe-to-bone test, imaging modalities (eg, plain radiograph or magnetic resonance imaging), inflammatory markers (erythrocyte sedimentation rate and C-reactive protein), and bone biopsy, is required to diagnose diabetic foot osteomyelitis (DFO).^10,40,41^ Along with a multidisciplinary approach to treatment, antibiotic selection is based on national guidelines and the best estimate of the local microbiological profile by clinicians.^30^

According to national guidelines, moderate to severe DFIs are treated with a range of antibiotics to cover aerobic and anaerobic bacteria (Table 1).

**Table 1.** Australian Therapeutic Guideline Recommendations for the Treatment of DFIs (Moderate and Severe).^a^

Severity of DFI	Antibiotic Recommendations According to National Guidelines (eTG)
**Moderate DFI**	Amoxicillin + clavulanate
	Immediate nonsevere or delayed nonsevere hypersensitive to penicillin Cefazolin + metronidazole For patients with immediate severe or delayed severe hypersensitivity to penicillin or who are at increased risk of MRSA, trimethoprim-sulfamethoxazole plus metronidazole If oral therapy is not an option, then ciprofloxacin and clindamycin/lincomycin can be used
**Severe DFI**	Piperacillin + tazobactam Hypersensitivity to penicillin, use ciprofloxacin plus either clindamycin or lincomycin Vancomycin—severe limb or life-threatening infection in patients at risk of MRSA infection

Abbreviations: DFI, diabetic foot infection; MRSA, methicillin-resistant *Staphylococcus aureus*.

a Adapted from *eTG Complete*.^12^

Microbial distribution underlying DFIs tends to follow a geographic pattern, and an improved understanding of this distribution can aid clinical decision making. *Staphylococcus aureus*, *Streptococci*, and *Enterococci* are the predominant gram-positive pathogens particularly in developed countries, whereas in developing countries, *Pseudomonas* spp, *Enterobacter* spp, and *Proteus* spp are the predominant gram-negative bacteria underlying DFIs.^21,48,51^

However, there is a scarcity of literature on the clinical demographics and microbial diversity of DFO in North Queensland, Australia. By providing an updated overview of the microbial diversity underlying DFO, we aim to help local clinicians tailor empirical therapy, prevent the overuse of antibiotics, and enhance insight into global antimicrobial susceptibility patterns.^2,24,26,48^

Hence, the aims of this study were threefold. Our first aim was to provide an overview of the incidence, clinical demographics, and glycaemic control of patients with DFO (as a complication of DFU) in tropical North Queensland. The second aim was to provide further insight into the microbial profile of DFO in our region and compare with global literature. The third aim was to evaluate whether national treatment guidelines align with the local antibiotic sensitivity profiles of bacterial isolates from bone cultures to guide future empirical therapy.

## Materials and Methods

A retrospective observational analysis was conducted with electronic clinical records from the high-risk foot clinic (an outpatient setting) and bone culture results from January 1, 2018, to December 31, 2022. Patients from the high-risk foot clinic were transferred to Townsville University Hospital (a tertiary referral hospital) for further treatment (eg, intravenous antibiotics).

The average incidence of DFO in this local region was calculated by dividing the average of the total cases of DFO and the general population of Townsville from 2018 to 2022. This value was then multiplied by 10 000 for a standardized value.

We focused on adult patients (aged >18 years) with type 2 diabetes associated with DFUs to ensure consistency with previous literature that demonstrated a higher prevalence of DFIs in this population.^5,7^ Bone samples were collected using strict aseptic techniques from the area that was likely to be infected on the basis of clinical signs and imaging modalities (either percutaneously or surgically). All bone biopsies were cultured for aerobic and anaerobic microorganisms and subjected to antibiotic sensitivity testing as per hospital protocols. Analysis and identification of pathogenic microorganisms were conducted by a certified microbiologist and laboratory staff at the study center. To determine causative and contaminant microorganisms, a methodical approach was applied with a semiquantitative analysis and determining if species identification aligned with the initial clinical presentation.

### Data Extraction Process

**Figure 1.**
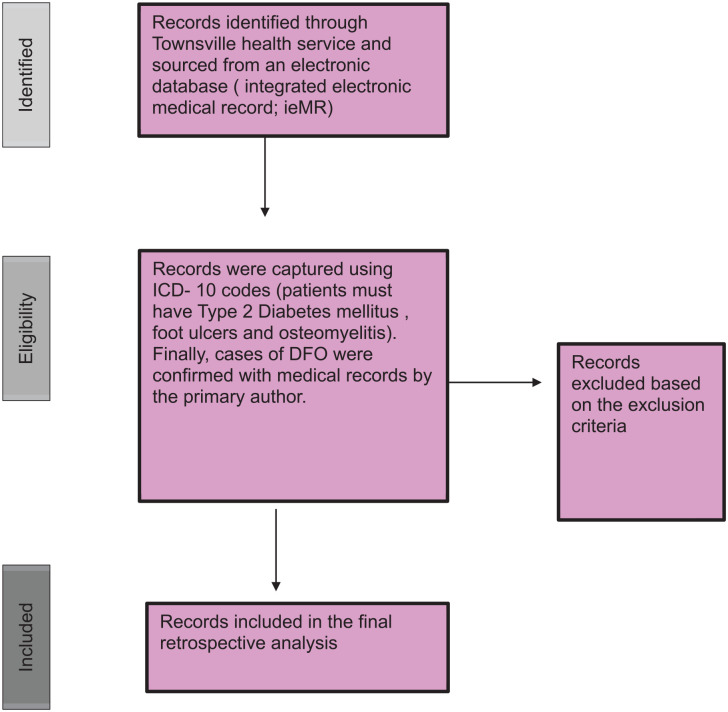
Detailed description of the data extraction and collection process. First, clinical records were identified from an electronic database. This was followed by data extraction and final retrospective analysis. *International Classification of Diseases, Tenth Revision*, codes were used.

Because of the confidential nature of the data, data extraction was performed by the research data laboratory team (Townsville Hospital and Health Service). Data were extracted from the Townsville Hospital and Health Service data warehouse using structured query language and Python code (Figure 1). All patients were sourced from an electronic medical record database (ieMR). To capture all cases, *International Classification of Diseases, Tenth Revision*, codes including “type 2 diabetes mellitus,” “foot ulcers” with complications, and “osteomyelitis of the ankle and foot” were used. Subsequently, the research data laboratory team extracted cohort demographics, in-scope comorbidities (chronic kidney disease, ischemic heart disease, and peripheral vascular disease), bone culture results, and HbA_1c_ levels. All data were accessible to the primary author via a secure network.

The primary author then excluded patients who were immunosuppressed as a result of medications/chemotherapy or diagnosed with nondiabetic causes of foot ulcers (eg, trauma, vasculitis, and neoplasms). The diagnosis of DFO was confirmed by a review of podiatry notes and bone culture results. Descriptions on DFUs including type and location were determined on a review of podiatry notes. Newly diagnosed DFO adhering to the study inclusion and exclusion criteria was considered for data analysis. Any missing data sets entailed casewise deletion from the analysis set.

### Statistical Analysis

All data were collated for analysis on an Excel sheet. SPSS, version 29.0 (IBM Corp, Armonk, NY), was used to analyze data. The frequency and percentages for variables, including sex, age groups, and comorbidities, were calculated. A χ^2^ test was performed to determine any association between Gram-positive or Gram-negative bacteria and age. The Fisher exact test results reported for cases if the assumptions for the minimum number required for the χ^2^ test were violated. A *P* value <.05 was considered to be statistically significant.

## Results

### Clinical Demographics

In this study cohort, 124 patients were diagnosed with DFO between January 1, 2018, and December 31, 2022. Non-Indigenous patients made up 68.5% of patients (n = 85), and Aboriginal and/or Torres Strait Islander people made up 31.5% (n = 39). Males (n = 87; 70.2%) and 50- to 69-year-old patients (n = 69; 55.6%) predominated this study cohort (Table 2). Most patients (53.3%) had HbA_1c_ levels >8.6% measured on admission. Ischemic heart disease, chronic kidney disease, and peripheral vascular disease were common comorbidities in 41.9%, 39.5%, and 33.1% patients, respectively (Table 2).

**Table 2.** Clinical Demographics of the Study Population and Descriptions of DFUs.^a^

Characteristic	n (%) or %
Ethnicity	
Non-Indigenous	85 (68.5)
Aboriginal and/or Torres Strait Islander people	39 (31.5)
Age, y	
<30	1 (0.8)
30-49	19 (15.3)
50-69	69 (55.6)
70-89	34 (27.5)
>90	1 (0.8)
Sex	
Male	87 (70.2)
Female	37 (29.8)
HbA_1c_	
<5.5-6.5	6 (4.8)
6.6-7.5	28 (22.6)
7.6-8.5	24 (19.4)
>8.6	66 (53.3)
Comorbidity	
Chronic kidney disease	49 (39.5)
Ischemic heart disease	52 (41.9)
Peripheral vascular disease	41 (33.1)
Anatomical location of DFU, %	
Forefoot	77.4
Midfoot or above	22.6
Lateralization of DFU, %	
Left	52.4
Right	47.6
Type of DFU, %	
Neuropathic	52.4
Neuroischemic	47.6

Abbreviation: DFU, diabetic foot ulcer.

a Most patients in this study tended to be non-Indigenous, males, and aged 50-69 years. Ischemic heart disease, chronic kidney disease, and peripheral vascular disease were comorbidities. In addition, DFUs tended to be located in the forefoot of the left lower limb and neuropathic in nature.

Neuropathic DFUs tended to be more common than neuroischemic ulcers (52.4% vs 47.6%, respectively; Table 2). A greater proportion of patients with DFUs were located on the left side (52.4%) and localized to the forefoot (ie, phalanges) compared with the midfoot or above (77.4% vs 22.6%, respectively; Table 2).

**Figure 2.**
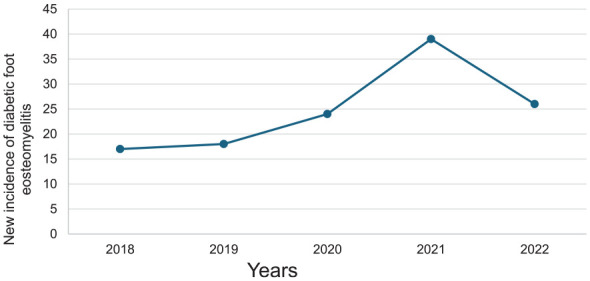
The incidence of diabetic foot osteomyelitis (DFO) over 5 years at the Townsville University Hospital between January 2018 and December 2022. From 2019 to 2021, the population incidence of DFO increased from 18 to 39 people. However, this decreased to 26 people in 2022.

In regard to the average incidence, 1.27 per 10 000 people were diagnosed with DFO in this population. From 2018 to 2021, the incidence of DFO increased from 17 to 39 cases (Figure 2), whereas from 2021 to 2022, the incidence of DFO decreased to 26 cases (Figure 2).

### Microbial diversity

**Figure 3.**
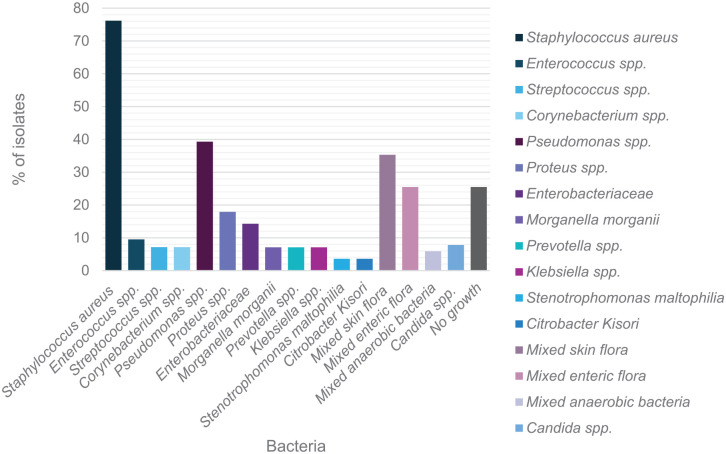
Distribution of microorganism isolates from bone cultures from patients with diabetic foot osteomyelitis from Townsville, Queensland. *Staphylococcus aureus* and *Pseudomonas* spp are predominant Gram-positive and Gram-negative species, respectively.

We identified 102 patients with bone culture results of resected bone. A total of 89 positive bone cultures were isolated including 65 (73.3%) monomicrobial cultures and 24 (24.9%) polymicrobial cultures (with at least 2 microbes). Gram-positive bacteria (n = 42) were the most common bacterial isolates. This was followed by Gram-negative bacteria (n = 28). The most common Gram-positive bacteria were *S aureus* (n = 32 [78.6%]; Figure 3) including methicillin-resistant *Staphylococcus aureus* (MRSA) (n = 11) and *Enterococci* spp (n = 4 [9.5%]; Figure 3). *Pseudomonas aeruginosa* was the most frequently isolated Gram-negative bacteria (n = 11 [39.3%]), followed by *Proteus* spp (n = 5 [17.9%]; Figure 3) and *Enterobacter* spp (n = 4 [14.3%]; Figure 3).

No growth, mixed skin flora, and enteric flora were represented in isolates (n = 13 [25.5%], n = 18 [35.3%], and n = 13 [25.5%], respectively; Figure 3). Notably, fungal and anaerobic bacterial isolates were also scarce in our study cohort (n = 4 [7.8%] and n = 3 [5.9%], respectively; Figure 3).

**Figure 4.**
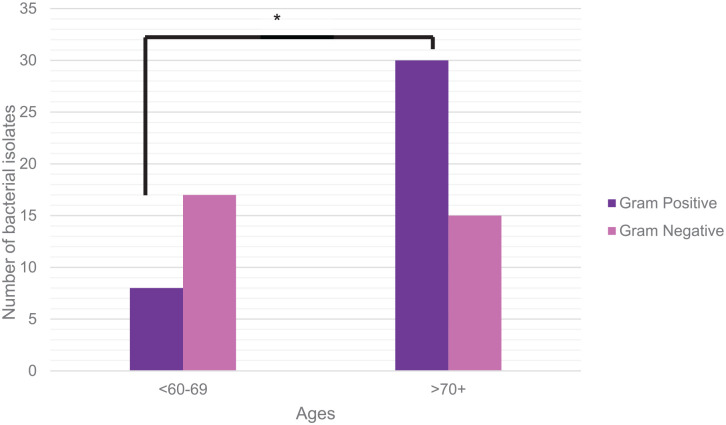
Gram-positive and Gram-negative bacteria analysis with age. Elderly patients (>70 years) diagnosed with diabetic foot osteomyelitis demonstrate a higher number of Gram-positive bacterial isolates compared with patients who are in the younger group (<69 years). In contrast, there was no statistical significance detected in the number of Gram-negative bacterial isolates between both age groups (*P < .05).

Gram-positive bacteria were found to be significantly increased in patients older than 70 years compared with those younger than 69 years (n = 30 vs n = 8; *P* < .05; Figure 4). The rates of Gram-negative bacteria isolated did not appear to differ between the 2 age groups (n = 17 vs n = 15; *P* > .05; Figure 4).

### Antibiotic Susceptibility Analysis

#### Gram-positive organisms

All MRSA isolates were sensitive to vancomycin and cotrimoxazole (100%; Table 3). In contrast, the susceptibility of MRSA isolates to clindamycin was moderately low (6 of 11 [54.5%]; Table 3).

**Table 3.** Sensitivity/Susceptibility Profile of Highly Prevalent Causative Pathogens Underlying Diabetic Foot Osteomyelitis to Relevant Antibiotics.^a^

Antibiotics	MRSA, % Susceptible (n) (n = 11)	*Pseudomonas aeruginosa*, % Susceptible (n) (n = 11)	*Proteus* spp, % Susceptible (n) (n = 5)
Vancomycin	100 (11)	–	–
Cotrimoxazole	100 (11)	–	100 (5)
Clindamycin	54.5 (6)	–	–
Piperacillin/tazobactam	–	72.7 (8)	–
Ciprofloxacin	–	18.2 (2)	–

Abbreviation: MRSA, methicillin-resistant *Staphylococcus aureus*.

a All MRSA isolates were sensitive to vancomycin (n = 11 [100%]). Both MRSA and *Proteus* spp were sensitive to cotrimoxazole (n = 11 [100%] and n = 5 [100%], respectively). In addition, *P aeruginosa* isolates were highly susceptible to piperacillin/tazobactam (n = 8 [72.7%]). Susceptibility to ciprofloxacin was low in *P aeruginosa* isolates (n = 2 [18.2%]).

#### Gram-negative organisms

Susceptibility of *Pseudomonas* spp isolates to piperacillin/tazobactam was generally high (8 of 11 [72.7%]; Table 3). Ciprofloxacin susceptibility was low in *Pseudomonas* spp isolates (2 of 11 [18.2%]; Table 3). All *Proteus* spp isolates were sensitive to cotrimoxazole (5 of 5 [100%]; Table 3).

## Discussion

This retrospective review provides a detailed overview of clinical demographics and the microbial data underlying DFO in North Queensland, Australia. Research detailing the microbiological diversity in patients with DFO is scarce in this region. Therefore, this study provides an updated overview of the microbiological profile in this unique geographical location.

In our study, DFO is relatively uncommon. Patients with DFO tend to be non-Indigenous males, aged 50-69 years, and with HbA_1c_ levels ≥8.6.

Monomicrobial isolates were predominant compared with polymicrobial cultures, and these results are in accordance with Álvaro-Afonso et al.^4^ This study aligned with findings from other developed countries, showing a higher prevalence of Gram-positive pathogens underlying DFIs, as determined by molecular and conventional culture methods.^30,47^ Conversely, in developing nations, a greater abundance of Gram-negative bacteria is observed in DFIs.^14,21,24,42^ This may be due to perianal washing after defecation in shared waterways, leading to exposure of gastrointestinal flora to ulcerations of the skin.^15^

Similarly, Wozniak et al investigating national geospatial surveillance data in Queensland demonstrated that up to 26% of skin swabs grew MRSA.^50^ The relatively high prevalence of MRSA is clinically significant in Queensland, because MRSA-related DFIs are associated with higher rates of osteomyelitis, intensive care unit admissions due to sepsis, and amputations.^5,11,32^ In contrast, no MRSA isolates were detected from a retrospective study of DFO conducted in a temperate setting in Scotland.^31^ Drawing on results described earlier, multiple etiologies may underlie our findings including geographical differences, humid weather leading to sweat on the skin promoting microbial growth, socioeconomic disparity, or differing antimicrobial use patterns.^13,18,31-33,45,51^

Our findings demonstrated that MRSA isolates were sensitive to vancomycin and cotrimoxazole, which are in accordance with other studies.^1,21^ Interestingly, clindamycin, an important second-line option to treat MRSA, demonstrated low susceptibility to MRSA in this study. This finding was in accordance with other literature, with resistance rates ranging from approximately 43.0% to 55.0%.^1,21^ This may be explained by cross-resistance from azithromycin use in the community to treat sexually transmitted infections, which has contributed to statewide resistance rates to clindamycin (20.0%).^9,50^ Hence, prudent use of antibiotics is recommended to prevent drug resistance.^20^

In our study cohort, *P aeruginosa* was the most commonly isolated Gram-negative bacteria. These findings are clinically significant because Gram-negative bacteria have been associated with increased risk of developing lower extremity amputations and mortality following DFIs.^19,29^ An example of this phenomenon may be explained by *P aeruginosa*, which promotes biofilm-mediated resistance and thrives in hypoxic environments found in deep bone infections,^19,43^ therefore worsening wound outcomes and leading to amputations. In our study, Gram-negative bacteria demonstrated a high sensitivity to recommended antibiotics, including piperacillin-tazobactam, for the treatment of severe DFIs. The high rate of susceptibility of Gram-negative bacteria to piperacillin-tazobactam has been documented by numerous studies.^4,16,21^ Resistance to ciprofloxacin in DFIs has been reported globally, including Italy, Egypt, and sub-Saharan Africa (ranging from 29.0% to 70.0%).^7,16,21,37,39,49^ Hence, caution is advised with ciprofloxacin use to treat DFO.

In this study, we found that Gram-positive organisms were significantly increased in older populations (≥70 years) compared with the younger age groups (<69 years). Indeed, older populations demonstrate an increased rate of MRSA colonization in soft tissue infections and a higher probability of DFIs positive for *S aureus*.^31,36^ The reason for this is unknown, but may be attributed to the association of MRSA with hospitalizations or persistent colonization following nursing home residence.^8,31^ Thus, with advanced age, patients acquire more risk factors for MRSA infection and should be screened for these risk factors to better guide the choice of empirical therapy.

Fungal and anaerobic isolates were scarce in our study. Molecular studies and conventional culture methods have indicated low prevalence of both these microbes in the context of DFIs.^3,23^ Our study adhered to laboratory protocols that use conventional culture methods. However, culture methods have limited capacity to identify anaerobic species and chronic biofilm infection and to provide a genomic map of bacteria.^22^ More sensitive molecular methods, including 16S rRNA subunit sequencing, will allow better identification of species and therefore refine the therapeutic management of DFO.^19,22^ Nonetheless, the clinical significance of fungal and anaerobic isolates in DFO merits further appreciation.^24,46,47^

The main limitation of this study lies in its retrospective nature. The primary investigator used electronic records for data analysis, which may lack relevant data for research. Prior antibiotics use in primary health care was unable to be accessed because of differing electronic databases in the community vs hospital setting. Next, our cohort size may have been impacted by pre- and post-lockdown periods during the COVID-19 pandemic (~2020-2022), because of disrupted access to health care.^44^ Although our sample size was relatively small, this study was designed to provide a snapshot of the problem. Nevertheless, it is unlikely that there would be significant deviations from the spectrum of organisms isolated from larger studies. Despite the inherent limitations that occur with a retrospective design, our study provides valuable microbiological surveillance and population data that will be paramount for enhancing clinical practice guidelines.

Because patients were referred from an outpatient setting to Townsville University Hospital for the treatment of DFO in this study, the microbiological analysis was performed with an assumption of community-onset DFO. For future research, any differences underlying the microbial profile of hospital-onset and community-onset DFO could be analyzed. Notably, national guidelines are used in both hospital and community settings for the treatment of DFO. However, hospital-acquired infections typically have higher rates of MRSA.^35^ Thus, investigating any differences in microbial profiles could tailor treatment strategies in either setting.

## Conclusion

This retrospective study has provided an updated overview of incidence, clinical demographics, bacterial spectrum, and data regarding antimicrobial resistance underlying DFO in North Queensland, Australia. Our results indicated a high prevalence of *S aureus* followed by *Pseudomonas* spp isolates in bone cultures from patients with DFO. On the basis of the findings from this study, the susceptibility results align with the antibiotics recommended in the national guidelines. Findings suggest that vancomycin and cotrimoxazole should continue to be used for MRSA infections. In addition, piperacillin-tazobactam may continue to be used for severe DFIs. Importantly, any antibiotic selection must be guided by microbiological evidence and relevant patient comorbidities including their renal and hepatic function. Lastly, this study demonstrated that Gram-positive bacteria are significantly associated with elderly patients with DFO. Knowledge of the microbial profile underlying DFO in North Queensland will assist in empirical therapy selection and offer global insight into antimicrobial susceptibility patterns.

## Supplemental Material

sj-pdf-1-fao-10.1177_24730114241281503 – Supplemental material for The Microbial Diversity and Antimicrobial Susceptibility Profile Underlying Diabetic Foot Osteomyelitis: A Retrospective Study Conducted in North Queensland, Australia Supplemental material, sj-pdf-1-fao-10.1177_24730114241281503 for The Microbial Diversity and Antimicrobial Susceptibility Profile Underlying Diabetic Foot Osteomyelitis: A Retrospective Study Conducted in North Queensland, Australia by Nandini Kulasegaran, Venkat Vangaveti, Robert Norton and Usman Malabu in Foot & Ankle Orthopaedics
